# Effects of Feeding and Maturation System on Qualitative Characteristics of Buffalo Meat (*Bubalus bubalis*)

**DOI:** 10.3390/ani10050899

**Published:** 2020-05-21

**Authors:** Raffaele Marrone, Angela Salzano, Antonio Di Francia, Lucia Vollano, Roberto Di Matteo, Anna Balestrieri, Aniello Anastasio, Carmela Maria Assunta Barone

**Affiliations:** 1 Department of Veterinary Medicine and Animal Production, University of Naples “Federico II”, 80137 Naples, Italy; raffaele.marrone@unina.it (R.M.); vollano@unina.it (L.V.); anastasi@unina.it (A.A.); 2 Department of Agricultural Sciences, University of Naples “Federico II”, 80055 Portici (NA), Italy; difranci@unina.it (A.D.F.); robdimat@unina.it (R.D.M.); cmbarone@unina.it (C.M.A.B.); 3 Istituto Zooprofilattico Sperimentale del Mezzogiorno, 80055 Portici (NA), Italy; a.balestrieri@yahoo.it

**Keywords:** colour, feeding, meat quality, post-ageing process

## Abstract

**Simple Summary:**

Nowadays, consumers used to being properly informed when purchased animal products. They are influenced by some meat characteristics like colour and tenderness. Nutrition is an important strategy to influence these properties, together with post day ageing processes. In this regard, the inclusion of fresh forage during fattening period and a prolonged maturation time in a monitored refrigeration device as Maturmeat^®^, were assessed on 16 Italian Mediterranean buffalo bulls. Data regarding physical-chemical analyses, colour and texture on two muscles, the *Semitendinosus* and the *Longissimus* muscles, were recorded during two maturation times (30 and 60 days). The combined used of fresh forage inclusion and post dry ageing processes influenced in a different way the two considered muscles, suggesting that different muscles could have different properties. Moreover, meat colour changed in both muscles in animals fed green forage and maturated in Maturmeat^®^ while no significant change was observed in the control group. In conclusion, the use of this monitored post dry ageing system could influence the saleable yield and productivity of buffalo meat and allow the creation of ready-to-eat food, produced starting from raw ground meat, that is very requested by the consumers.

**Abstract:**

We aimed to evaluate the effects of post dry ageing (PDA) period on meat colour and rheological characteristics in 16 buffalo bulls fed two different diets: with (FRS) or without (CTL) rye grass. Animals were randomly divided into two feeding groups and slaughtered at 540 ± 4.7 and 533 ± 7.0 kg of live body weight, respectively, for the CTL and FRS group. After five days post-mortem ageing (T0), *Semitendinosus* muscle (ST) and *Longissimus* muscle (LD) underwent a prolonged maturation process in a controlled meat chamber for 30 days (ST) and until 60 days (LD). After 30 days (T1), significant changes (*p* < 0.01) in meat colour (ΔE) in both muscles of the FRS group was recorded, while no significant change was observed in CTL group. The FRS diet had a positive effect on textural properties of ST muscle compared to CTL diet, as well as hardness, chewiness and gumminess. All qualitative characteristics improved in the first period of PDA but, whereas LD showed to keep improving, extending the post-ageing period by further 30 days, the ST becomes un-processable at 60 days. In conclusion, a combined used of fresh feeding and PDA period could enhance both tenderness and colour in animal fed FSR.

## 1. Introduction

Meat is a good source of nutrients for human beings and provides high quality protein, fats, minerals and vitamins [1]. Red meat particularly, is a good source of zinc, iron, selenium, calcium, phosphorus and lipids, followed by vitamin A and B-complex vitamins [2]. Recently, it has been seen that consumers are more attracted in unprocessed food, such as raw ground meat (ex. tartare) or rare cooked meat steak, and tenderness, colour and freshness are considered to be among the most important value-determining factors affecting consumers’ meat purchasing decision [3,4,5]. Although meat colour is the attribute that most influences punctual purchasing decisions, because it is the only trait they can evaluate at the point of sale [6,7], most of the repeated purchases behaviour is often dependent on meat tenderness. Buffalo meat could be a valid alternative to beef [8], since the nutritional and organoleptic characteristics of buffalo meat ensure a high dietary value and make it suitable to be included in the Mediterranean diet, in case of personalized nutritional health needs [2]. Buffalo breeding in the world has been encouraged because of the ability of the species to adapt to various climatic conditions, greater digestibility of poor-quality pasture, and faster growth, making it a versatile and useful species for sustainable livestock production [9]. In terms of composition, quality, and organoleptic characteristics, Mediterranean buffalo meat is almost similar to cattle meat of the same age [10]. Moreover, it is appreciated for its lower fat, cholesterol, and calory content [11], and for its positive effects on human cardiovascular risk profile [12]. Although several studies have been carried out on ageing system and quality of beef [13,14,15], few studies are available on buffalo meat quality [16] and particularly on the ageing process [17]. This is because in Italy, buffalo breeding was primarily focused on milk production, while only recently Italian stockbreeders focused also on meat. In order to meet market demands and breed high-quality standard animals, several studies showed the influence of feeding on carcass quality and on meat characteristics. According to de la Cruz-Cruz et al. [18], feeding system and microclimate deeply affect both buffalo welfare and quantitative and qualitative meat production. Negative meat traits for consumers are dark colour, low marbling and low tenderness [6], and the first two characteristics are typical of buffalo meat.

Substantial improvements in meat palatability attributes such as tenderness, flavour, and/or juiciness could be obtained with an ageing processes [19]. The impact of ageing and following tenderization on meat quality depends on many factors such as species, animal age, diet, breed, type of muscle, marbling characteristic and ageing conditions [20]. Innovative concepts regarding beef ageing has been described in many studies [17,21]. These systems improve post-mortem proteolysis and calpains myofibrillar processing that both have an important role in influencing meat tenderness and represent a marker of meat quality [22]. It has also been seen that grass-fed beef has some potential health benefits, mainly due to the nature and amount of fat deposited in the lean [23]. The results of Apaoblaza [24] showed that L* and a* values, which describe the intensity of whiteness/brightness and red colour respectively, are lower in grass-fed beef compared to grain-fed beef, while Lambertz et al. [25], observed no effects on L* and higher a* value in buffalo concentrate-fed rather than pasture-fed. Therefore, the aim of the study was to determine the combinate effect of including fresh forage in the diet with an innovative post dry ageing (PDA) process on rheological parameters and the colour of Mediterranean buffalo meat.

## 2. Materials and Methods 

### 2.1. Animals and Diets

The study was carried out in a commercial buffalo dairy farm (Verdi Praterie, Kr, Italy) located in southern Italy on 16 Italian Mediterranean buffalo bulls with an average age of 581 ± 0.4 days and an average weight of 460 ± 3.5 kg. Buffaloes were kept at feedlot and randomly divided into two groups: with (fresh group, FRS; n = 8) or without (dry group, CTL; n = 8) the inclusion of rye grass for three months (fattening period). The amount and the composition of the diets are reported in Table 1. Animals were slaughtered at 540 ± 4.7 and 533 ± 7.0 kg of live body weight in CTL and FRS groups, respectively, and all carcasses had a grade U2 according to E.U.R.O.P. conformation (classification: five classes, from E = “excellent”, U = “very good”, R = “good”, O = “fair” to P = “bad” conformation) and fatness score (five classes, from 1 = lean, to 5 = fat).

The animals were slaughtered in an EU-approved slaughterhouse by exsanguination after stunning by captive bolt and carcasses were dressed without any electrical inputs and chilled at 4 °C. After five days post-mortem ageing (T0), a section of Longissimus muscle (for a total of seven vertebrae, starting from the sixth to the last thoracic vertebrae, LD) and a central portion (about 30 cm) of Semitendinosus muscle (ST) were removed from the carcasses and placed into the MaturMeat^®^ (European Patented Device and meat Dry Aging Method with safe and controlled Ph - n. EP 2769276B1) for post dry ageing (PDA). The Maturmeat^®^ is a monitored refrigeration device in which the operator can customize ageing conditions, such as temperature and relative humidity. This device had an inner control system that set the relative humidity by the injection of water vapour until a pre-set percentage, and a control system that recorded these variables. In this way, the operator was able to recover all the information and to control the parameters. For this experiment, the temperature and humidity were set to 2 °C and 78%, respectively. The buffalo meat cuts were placed on steel grids and monitored constantly to avoid mould growing or any external font of variability. Samples were weighed and each length dimension was registered at each experimental time (T0 = 5 d ageing, T1 = 30 d post ageing; T2 = 60 d post ageing), in order to control the data during the time and to measure the weight loss and shrinkage rate, respectively. After 30 days of post ageing (T1), meat was taken out of the MaturMeat^®^ chamber and sampled for the analysis. At the end of maturation period (T2), ST portions were not analysable because too dark and dry to be measured, so we considered 30 days for both muscles and 60 days only for the LD muscle.

### 2.2. Physical-Chemical Analyses

Physical-chemical analyses were evaluated at each time and in both muscles by pH, measured with a digital pH meter (Crison-Micro TT 2022, Crison Instruments, Barcelona), by activity water (aw) (Aqualab 4 TE - Decagon Devices Inc., USA). The moisture (%) was determined on 100 g of meat by oven drying for 24 h at 105 °C [26].

### 2.3. Colour 

Meat colour parameters L* (lightness), a* (redness) and b* (yellowness) were measured using a Konica Minolta CM-2500d (Konica Minolta Sensing Inc., Osaka, Japan) with an 8-mm diameter measuring aperture, calibrated against a white standard plate. The operative conditions were Illuminant D65 and 10° standard observer. Value of chroma (C*) and hue angle (h°) were calculated using Equations (1) and (2), respectively:

(1) $$C^{*}=\sqrt{a^{*2}+b^{*2}}$$

(2) $$h{}^{\circ}=arctan\frac{b*}{a*}$$

At the beginning (T0), in the middle (T1) and at the end (T2) of the post ageing period, colour measurements of the muscles were assessed by three different locations on the fresh cut. Changes in colour during post ageing were determined by the colour differences coefficient (ΔE) between the initial (T0) and final (T1 for ST and T2 for LD) colour of the samples, calculated from Equation (3):

(3) $$\Delta E^{*}=\sqrt{{\left(\;L^{*}{}_{\;end}-L_{0}^{*}\right)}^{2}+{\left(a^{*}{}_{end}-a^{*}{}_{0}\right)}^{2}+{\left(b^{*}{}_{end}-b^{*}{}_{0}\right)}^{2}}$$

### 2.4. Warner-Bratzler Shear Force

Seven cores (1.27 cm in diameter and 2.5–3 cm in length) were obtained using a well-sharpened hand-hold coring device that was oriented parallel to the longitudinal orientation of muscle fibres. The shearing force was applied perpendicular to fibre orientation using Instron Mod. 5565 equipped with a V-shaped shear blade (load 500 kg, head speed 200 mm/min). The mean value of the seven cuts was used for statistical analyses. The measured parameters were Myofibrillar Shear Force (MSF) and Warner-Bratzler Shear Force (WBSF) both expressed in kg, according to Cimmino et al. [27].

### 2.5. Texture Profile Analysis

This test measures the compression force (Newtons) developed by the texturometer (EZ-Test Shimadzu), Shimadzu Corporation, Japan) when compressing a piece of meat. A cylindrical 25-mm diameter probe was used for all texture profile analysis (TPA) in this study. Each sample was placed under the probe that moved downwards at a constant speed of 50 mm s-1. The probe continued downwards on a pre-fixed percentage of the sample thickness (80%). During the test run, the resistance of the sample was recorded every 0.01 s and plotted in a force-time (grams-seconds) plot in order to have the following parameters [28]: hardness (maximum force applied), cohesion (strength of internal bonds measured by ratio between two consecutive compressions), elasticity (rate of return after compression), gumminess (hardness x cohesion) chewiness (gumminess x elasticity), resilience (similar to elasticity but expressed as a ratio of energies), adhesivity (work needed to overcome the forces between the sample and the probe). Each measurement was assessed 7–10 times and the average values were used for statistical analysis.

### 2.6. Statistical Analyses

The animal was the experimental unit. All data were analysed performing ANOVA for each muscle (ST and LD), by using the mixed model of SAS software package [29]) with feeding system (diet without rye grass, CTL; diet with rye grass, FRS) as non-repeated factor and time (T0 = 5 d of ageing, T1 = 30d post ageing, T2 = 60d post ageing only for LD) and interaction considered as repeated factors. The animal was considered as random effect and its variance was utilised as error term to test the main effect of the feeding system. All data were presented as the least square mean ± standard error (SEM). To evaluate differences among means, Tukey’s test was performed for each significant effect (*p* < 0.05).

## 3. Results and Discussion 

### 3.1. Effects of Feeding 

The daily average weight gain was 849 ± 12.0 and 838 ± 9.2 g, respectively, for CTL and FRS group for the fattening period (90 days). At slaughter, live body weights were 540 ± 4.7 and 533 ± 7.0 kg in CTL and FRS groups, respectively. No differences were recorded in terms of carcass weight (kg) between CTL and FRS groups (273.7 ± 2.4 vs 271.8 ± 3.4, respectively, in CTL and FRS groups). The SEUROP score for conformation was R2 class and the average carcass yield of 51 % in both groups. The analysis of variance showed that the diet interacts with the post ageing process in determining the colour attributes in both studied muscles (Table 2). This interactive effect gave a significant improvement in the colour of both muscles after 30 days of post ageing (T1) in the FRS group (*p* <0.01), showing lower value of redness index (a*) and chroma (less brightness) and greater L* value (meat was lighter), whereas no significant change in the CTL group (Table 2) was determined. Extending the post ageing period, the instrumental colour traits of LD did not change. It should also be noted that the FRS meat already started (T0) with higher values (*p* < 0.01) of a*, b*, chroma and hue angle and lower lightness (*p* < 0.01), compared to CTL meat, but at 30 days of post ageing, the same meat showed lower yellowness (b* values, *p* < 0.01) and a concomitant lower hue angle (*p* < 0.01) that makes it less brown in both muscles (Table 2).

**Figure 1:**
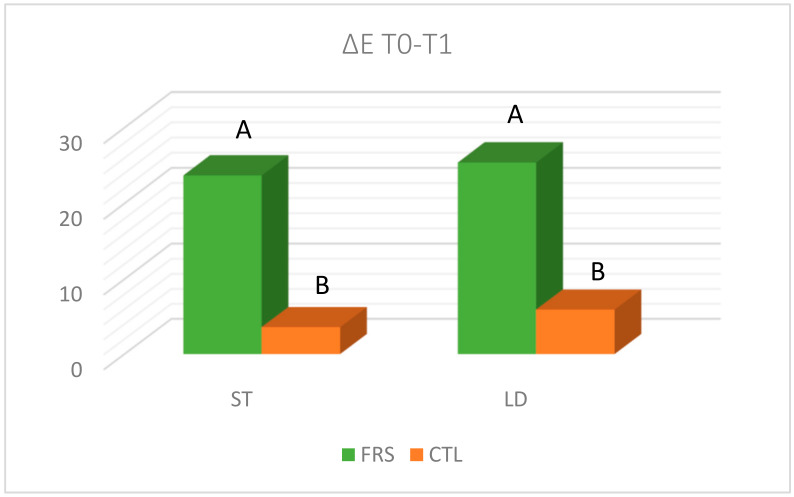
Variation of colour (ΔE) during the first 30 days (T0-T1) of PDA in buffaloes fed with (FRS) or without (CTL) rye grass in *Semitendinosus* (ST) and *Longissimus* (LD) muscle. ^A, B,^ Bars with different letters are significantly different; *p* < 0.01

Many researchers reported that pasture diets were responsible for darker meat in comparison to concentrate diets [30,31]. Buffalo meat is darker than beef, and this is one of the factors that often, although unconsciously, could influence the refusal to purchase this product. The effects of the feeding system basically displayed a more stable colour in the CTL compared to FRS group, also evaluated by the delta E (ΔE) value, which measures total colour change of combined changes in L*, a*, and b* (Figure 1). Dosi et al. [32] found that the amino acid sequence of buffalo myoglobin, that is different from bovine for the presence of negatively charged residues at some specific positions (19 in helix A and 117 in helix G), could be responsible for the rapid discolouration of buffalo meat. Our findings partially disagree with Dosi et al. [32] because in the CTL group the meat colour remained constant during the time for both muscles. One of the peculiarities of buffalo meat is the high concentration of iron and unsaturated fatty acids that promote an earlier darkening compared to beef [33]. This problem could be overcome by the administration of vitamin E in the diet (1500 IU/die; cost of treatment 0.86 EUR/kg of meat) that increases colour stability making meat more “pleasing” (lighter) and still saleable eleven days after slaughter [33], probably for an estimated lower metmyoglobin content [34]. 

**Figure 2:**
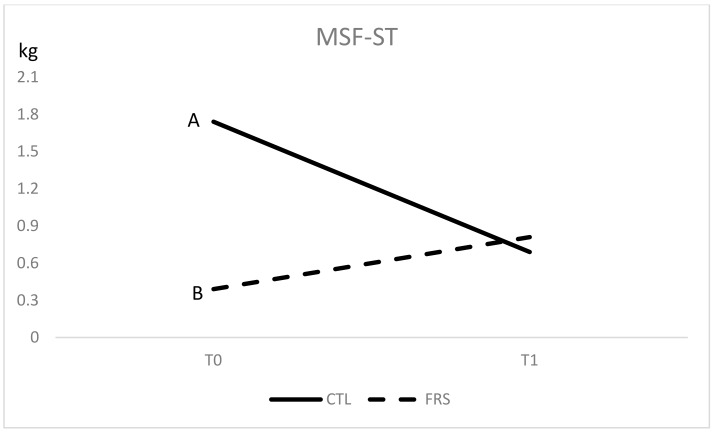
Myofibrillar shear force (MSF) of *Semitendinosus* muscle (ST). CTL = diet without rye grass; FRS = diet with rye grass; T0 = 5 days of ageing, T1 = 30 days post ageing. A, B = *p* < 0.01.

Considering beef, when ΔE is >0.9 the difference in meat colour is visually detectible and also useful for differentiating the pH of the meat [35]. As reported by Neethling et al. [36], colour stability can be affected by the feeding management involving extensive (grass/pasture/forage) or intensive (concentrate/grain/feedlot) systems. In this study, inferring the feeding effect is problematic due to the interaction, although the estimated mean values independently from PDA factor, showed that FRS diet determined lower whiteness/brightness value (L*), in comparison to CTL diet (32.6 and 36.2, SEM 1.0 *p* < 0.05, respectively), even if only in the LD muscle. Moreover, the probably higher level of carotene in the FRS diet would be responsible for the higher b* values (14.9 vs 11.1, SEM 0.6 *p* < 0.05), whereas the higher a* value (17.2 vs 14.6, SEM 0.6; *p* < 0.05) contradicts the results of Lambertz et al. [25], which found redder meat in buffalo who received concentrate in comparison to pasture. Textural traits measured by the Warner-Bratzler test showed a significant interaction feeding system x PDA only for myofibrillar shear force (MSF) of ST muscle which decreased from 1.7 to 0.7 kg in the first post-ageing period (from T0 to T1) in CTL group (*p* < 0.01) whereas did not change significantly in fresh fed group (Figure 2). This last group showed a significant lower level of MSF, with respect to CTL (*p* < 0.01), at T0.

Regarding the parameters detected by the texture profile analysis, while no significant differences were observed between feeding system for LD muscle (Table 3), there was a positive effect of FRS diet on ST muscle as animals of this group displayed lower tenderness, chewiness and gumminess values (*p* < 0.01) than CTL group (Table 4).

### 3.2. Effects of Post Dry Ageing 

The effects of post dry ageing, independently from diet, on physical-chemical characteristics of meat are reported in Table 3 and Table 4. Both pH and aw did not change, showing similar behaviour in the two muscles, according to previous studies in beef [37,38]. On the contrary, post dry ageing affected weight loss in both groups in the first 30 days. According to Velotto et al. [39], this could be due to the loss in moisture percentage showed (69.4 to 62.4 SEM 0.7 in LD muscle and 72.5 to 67.82 SEM 0.6 in ST muscle) (*p* < 0.01) and trimming waste. During PDA, meat colour changed in different ways in the studied muscles; in the first 30 days a*, b* chroma and hue angle in both groups decreased significantly while lightness increased (data not showed), but, extending the period by further 30 days, the LD colour did not change significantly while ST became too dark and dry to be measured. Therefore, during the first 30 days, meat became lighter and more pink-gray for both muscles. These results underline that a post ageing period of 30 days would make possible to obtain a “clearer” colour, making buffalo meat visually more acceptable. Ramanathan et al. [40] report that an extended ageing time (>14 days) decreases colour stability, while it has a limited benefit on improving tenderness. However, in our study we found out that after 30 days of post dry ageing, while CTL group maintained a colour stability, in FRS group meat became lighter. Regarding rheological properties, the PDA process decrease hardness, gumminess and chewiness, even if significantly only in LD muscle (Table 4).

Indeed, hardness and related parameters, gumminess and chewiness, decreased (*p* < 0.05) more than 60% and resilience about 31% (*p* < 0.05) in LD during the first 30 days of PDA. Different levels of tenderness were reported by Wheeler et al. [41] on distinct bovine cuts. On several muscles of buffalo and bovine calves slaughtered at 20, 28 and 36 weeks of age, also Matassino et al. [10] showed that “the muscle has marked individuality” in rheological, chemical and colour traits. This could suggest different procedure of post ageing treatment for the different cuts in buffalo carcass and that the use of the only LD muscle may not fully representative of the physiological and rheological condition of the carcass.

## 4. Conclusions

Colour and tenderness are critical to the economic competitiveness of buffalo meat industry as consumers use them as their best indicator for wholesomeness. The interactive effect between diet and post ageing process improved the development of colour and tenderness of both muscles in the fresh group, showing in particular a lower value of redness index, greater lightness value and lower myofibrillar shear force. This study proposed a new joint approach with both fresh diet and PDA process in a monitored refrigeration device, such as Maturmeat^®^, in order to improve the general aspect and texture of buffalo meat. This new method had a significant effect on texture measurements in LD muscle, and colour improvement in FRS meat, influencing the saleable yield and productivity of buffalo meat. Moreover, the use of this monitored PDA system could allow the creation of ready-to-eat food, produced starting from raw ground meat, that is very requested by the consumers.

## Figures and Tables

**Table 1:** Feed (kg) and chemical composition (% of dry matter, DM) of the diet in buffaloes with (FRS) or without (CTL) rye grass inclusion.

Feed (kg)	Diet	
	CTL	FRS
Rye grass	-	12
Straw	3	2
Concentrate	7	5
Calcium carbonate	-	0.1
Water	8	-
TOTAL	18.0	19.1
	**Nutritive Value**	
MFU/kg DM	0.93	0.93
	**Chemical Composition**	
Dry Matter (kg)	8.6	8.6
Dry matter (%)	47.7	44.7
Moisture (%)	52.3	55.3
Crude protein (CP)	12.3	12.2
NDF (%)	36.0	36.1
ADF (%)	16.0	19.4
ADL (%)	5.0	5.4
Ca (%)	0.7	0.7
P (%)	0.4	0.4

MFU = Meat Forage Unit; NDF = Neutral Detergent Fibre; ADF = Acid Detergent Fibre; ADL = Acid Detergent Lignin.

**Table 2:** Effects ^1^ of interaction feeding system x post ageing time on meat colour in buffaloes fed with (FRS; n = 8) or without (CTL; n = 8) rye grass. Mean value and standard error of the mean (SEM).

Trait	Diet	Post Ageing			SEM	Pr>F	Post Ageing		SEM	Pr > F
		T0	T1	T2			T0	T1		
		*Longissimus*					*Semitendinosus*			
Lightness, L*	CTL	35.61	35.49	37.57	1.36	0.0004	36.72	35.40	1.42	0.0008
	FRS	25.33^B^	37.28^A^	35.12^A^			26.90^B^	37.56^A^		
		***					**			
Redness, a*	CTL	14.58	14.99	14.15	1.15	0.0041	12.86	13.97	0.60	<0.0001
	FRS	22.98^A^	13.53^B^	15.22^B^			22.92^B^	13.40^A^		
		***					***			
Yellowness, b*	CTL	10.25	12.85	10.11	1.02	<0.0001	11.75	13.60	0.68	<0.0001
	FRS	27.14^A^	7.14^B^	10.39^B^			28.14^B^	9.47^A^		
		***	**				***	**		
Chroma	CTL	17.83	19.81	17.39	1.33	<0.0001	17.42	19.54	0.72	<0.0001
	FRS	35.64^A^	15.35^B^	18.44^B^			36.32^B^	16.41^A^		
		***					***			
Hue angle	CTL	35.08	39.92	35.46	2.07	<0.0001	42.42	43.97	1.32	0.0003
	FRS	49.95^A^	28.11^B^	34.30^B^			50.92^A^	35.27^B^		
										
		***	**				**	**		

^1^ T0 = 5 d ageing, T1 = 30 d post ageing; T2 = 60 d post ageing. Means with different letters within a row (post ageing time) differ significantly for *p* < 0.05 (small) or *p* < 0.01 (capital) while different symbols within a column indicate that means differ significantly for *p* < 0.01 (**) or p < 0.001 (***).

**Table 3:** Effect of feeding system and post dry ageing on meat quality of buffaloes fed with (FRS; n = 8) or without (CTL; n = 8) ray grass in *Longissimus* muscle.

Parameter	Feeding System		SEM	Pr > F	Post Ageing			SEM	Pr > F
	CTL	FRS			T0	T1	T2		
pH	5.73	5.73	0.03	0.9176	5.57	5.66	5.72	0.05	0.9010
Aw	0.981	0.982	0.001	0.2735	0.981	0.978	0.982	0.001	0.2521
**Texture**									
MSF, kg	0.73	0.63	0.22	0.7693	0.44	0.68	0.92	0.21	0.3034
WBSF, kg	4.9	4.76	0.27	0.7596	4.00	5.00	5.50	0.42	0.1023
Hardness, N	14.26	9.5	3.12	0.3224	19.86^a^	7.67^b^	8.12^b^	3.18	0.0173
Gumminess	5.98	4.1	1.39	0.3756	8.73^a^	3.23^b^	3.13^b^	1.42	0.0150
Chewiness	3.92	2.75	0.95	0.4181	6.00^a^	2.04^b^	1.96^b^	0.96	0.0104
Elasticity, cm	0.66	0.64	0.02	0.4360	0.69	0.64	0.63	0.02	0.0618
Resilience	0.14	0.12	0.01	0.4349	0.16^a^	0.11^b^	0.13^b^	0.01	0.0403
Cohesion	0.41	0.41	0.01	0.7838	0.44	0.42	0.37	0.02	0.0243
Adhesivity	-0.6	-0.37	0.18	0.3886	-0.59	-0.32	-0.55	0.21	0.6211

Aw = Activity water; MSF = Myofibrillar Shear Force; WBSF = Warner-Bratzler Shear Force. Data are reported as average values ± SEM. T0 = 5 d ageing, T1 = 30 d post ageing; T1= 60 d post ageing. ^A, B,^ Values in the same row with different letters are significantly different (*p* < 0.05).

**Table 4:** Effect of feeding system and post dry ageing on meat quality of buffaloes fed with (FRS; n = 8) or without (CTL; n = 8) ray grass in *Semitendinosus* muscle.

Parameter	Feeding System		SEM	Pr > F	Post Ageing		SEM	Pr > F
	CTL	FRS			T0	T1		
pH	5.71	5.75	0.015	0.2592	5.56	5.64	0.014	0.2460
Aw	0.978	0.981	0.001	0.1381	0.981	0.977	0.001	0.0651
**Texture**								
MSF, kg	1.21^A^	0.60^B^	0.21	0.0078	1.82^A^	0.75^B^	0.18	0.0033
WBSF, kg	7.47	7.52	0.47	0.9462	7.18	7.81	0.49	0.4080
Hardness, N	48.89^A^	19.18^B^	3.8	0.0015	36.72	31.35	4.46	0.4790
Gumminess	16.68^A^	8.60^B^	1.34	0,005	13.74	11.54	1.5	0.3811
Chewiness	12.14^A^	5.94^B^	0.97	0.0041	9.69	8.39	1.09	0.4718
Elasticity, cm	0.74	0.73	0.02	0.5625	0.72	0.75	0.02	0.2693
Resilience	0.23	0.19	0.02	0.1939	0.19	0.22	0.02	0.3490
Cohesion	0.36^A^	0.52^B^	0.03	0.0049	0.43	0.46	0.02	0.3231
Adhesivity	-0.47	-1.38	0.29	0.0680	-0.78	-1.07	0.31	0.5586

Aw= Activity water; MSF = Myofibrillar Shear Force; WBSF = Warner-Bratzler Shear Force. Data are reported as average values ± SEM. T0 = 5 d ageing, T1 = 30 d post ageing; T1 = 60 d post ageing. ^A, B,^ Values in the same row with different letters are significantly different (*p* < 0.01).
